# A semi-automated measuring system of brain diffusion and perfusion magnetic resonance imaging abnormalities in patients with multiple sclerosis based on the integration of coregistration and tissue segmentation procedures

**DOI:** 10.1186/s12880-016-0108-1

**Published:** 2016-01-14

**Authors:** Alfredo Revenaz, Massimiliano Ruggeri, Marcella Laganà, Niels Bergsland, Elisabetta Groppo, Marco Rovaris, Enrico Fainardi

**Affiliations:** Unità Operativa di Neuroradiologia, Dipartimento di Neuroscienze e Riabilitazione, Azienda Ospedaliero-Universitaria of Ferrara, Arcispedale S. Anna, Via Aldo Moro 8, 44124 Cona, Ferrara, Italy; Consiglio Nazionale delle Ricerche, 44124 Ferrara, Italy; MR Research Laboratory, IRCCS Don Gnocchi Foundation ONLUS, Milan, Italy; Buffalo Neuroimaging Analysis Center, Department of Neurology, University at Buffalo SUNY, Buffalo, NY USA; Sezione di Neurologia, Dipartimento di Scienze Biomediche e Chirurgico Specialistiche, Università di Ferrara, Ferrara, Italy; Unità Operativa di Sclerosi Multipla, Fondazione Don Gnocchi ONLUS, IRCCS S. Maria Nascente, 20148 Milano, Italy

**Keywords:** DPP Suite, Coregistration, Automatic segmentation, Automatic classification, DWI, PWI

## Abstract

**Background:**

Diffusion-weighted imaging (DWI) and perfusion-weighted imaging (PWI) abnormalities in patients with multiple sclerosis (MS) are currently measured by a complex combination of separate procedures. Therefore, the purpose of this study was to provide a reliable method for reducing analysis complexity and obtaining reproducible results.

**Methods:**

We implemented a semi-automated measuring system in which different well-known software components for magnetic resonance imaging (MRI) analysis are integrated to obtain reliable measurements of DWI and PWI disturbances in MS.

**Results:**

We generated the Diffusion/Perfusion Project (DPP) Suite, in which a series of external software programs are managed and harmonically and hierarchically incorporated by in-house developed Matlab software to perform the following processes: 1) image pre-processing, including imaging data anonymization and conversion from DICOM to Nifti format; 2) co-registration of 2D and 3D non-enhanced and Gd-enhanced T1-weighted images in fluid-attenuated inversion recovery (FLAIR) space; 3) lesion segmentation and classification, in which FLAIR lesions are at first segmented and then categorized according to their presumed evolution; 4) co-registration of segmented FLAIR lesion in T1 space to obtain the FLAIR lesion mask in the T1 space; 5) normal appearing tissue segmentation, in which T1 lesion mask is used to segment basal ganglia/thalami, normal appearing grey matter (NAGM) and normal appearing white matter (NAWM); 6) DWI and PWI map generation; 7) co-registration of basal ganglia/thalami, NAGM, NAWM, DWI and PWI maps in previously segmented FLAIR space; 8) data analysis. All these steps are automatic, except for lesion segmentation and classification.

**Conclusion:**

We developed a promising method to limit misclassifications and user errors, providing clinical researchers with a practical and reproducible tool to measure DWI and PWI changes in MS.

## Background

The role of diffusion-weighted imaging (DWI) and perfusion-weighted imaging (PWI) modalities in multiple sclerosis (MS) has recently received increased attention due to the potential of these two advanced magnetic resonance imaging (MRI) techniques in detecting the structural and hemodynamic characteristics of MS-related focal [1–7] and diffuse [8–16] brain abnormalities in gray and white matter, which characterize the well-known heterogeneity of the disease [17]. In fact, although conventional MRI findings are currently considered a valid surrogate marker for MS diagnosis and progression in treated and untreated patients, their diagnostic and prognostic value still remains very limited given the inability of conventional MRI in identifying the specific pathologic substrates of MS lesions [17]. Thus, it could be crucial to understand whether the detection and quantification of focal and diffuse DWI and PWI changes may help in recognizing the different mechanisms implicated in MS damage and, as a consequence, in improving diagnostic accuracy, early outcome prediction and response to treatment monitoring in MS. As DWI and PWI can be easily integrated in the context of MRI examination, this may have a large impact for routine clinical setting and patient quality of life. Therefore, further studies are warranted to clarify the actual significance of DWI and PWI disturbances in MS. However, the evaluation of DWI and PWI alterations in MS is generally restricted to the research field and is currently performed by different software programs, used separately from each other with lack of standardization regarding the overall process [1–16, 18, 19]. This implies several steps including various co-registration and tissue/lesion segmentation tasks which make the analysis rather laborious, time-consuming and prone to inaccuracies due to human intervention. These limitations could explain why the results coming from previous studies were not always concordant. In addition, there are no large-scale studies investigating DWI and PWI abnormalities in MS focal lesions categorized according to different stages of their evolution (acute and chronic) [20]. Thus, a more integrated analysis process incorporating all software packages employed in performing co-registration and tissue/lesion segmentation steps would be beneficial. This approach should be based on the activation of the different software modules according to a logical sequence leading to precise measurements of DWI and PWI values in focal MS lesions, in normal appearing grey matter (NAGM) and in normal appearing white matter (NAWM). For these reasons, the pipeline proposed in this work consists in an implementation of already described algorithms [4, 6, 10, 13, 15] combined according to a hierarchical order in a semi-automated manner. Although many automated methods have recently been developed for MS lesion detection [21–23], we chose semi-automated operations for the identification of focal damage to minimize potential biases due to lesion misclassification [18, 19], which could overcome operator-dependent misinterpretations related to visual inspection. On the other hand, the lack of clear standards renders it difficult to judge the superiority of one approach over other available choices [18, 19, 24]. Accordingly, we included tools commonly used in the MS literature which have been demonstrated to be both efficient and accurate in co-registration and tissue/lesion segmentation procedures. Moreover, the investigators of our group have many combined years of experience using these tools [18, 19, 24–26]. In this way, we sought to provide a promising tool for reducing analysis complexity and obtaining reproducible results.

## Methods

### Definition and general description of the DPP Suite and modules

**Fig. 1:**
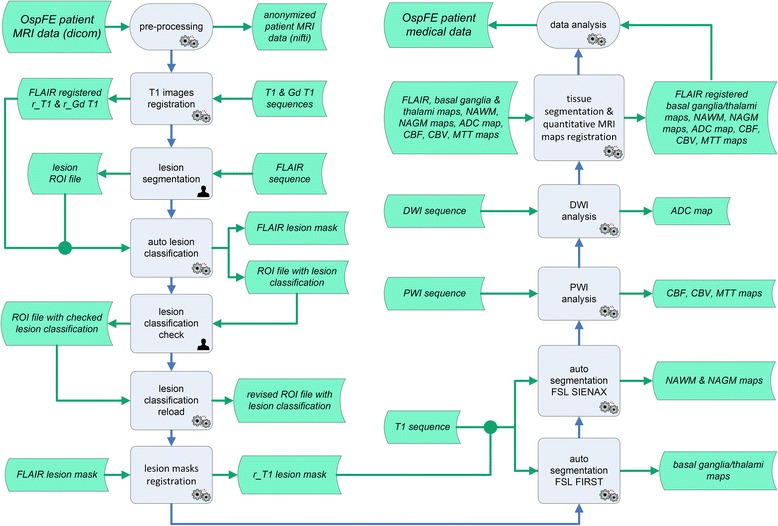
Flowchart of Diffusion/Perfusion Project (DPP) Suite. The DPP Suite is used for diffusion-weighted imaging (DWI) and perfusion-weighted imaging (PWI) measurements in focal and diffuse abnormalities in multiple sclerosis (MS) patients. OspFE = Ferrara Hospital; MRI = magnetic resonance imaging; FLAIR = axial fluid-attenuated inversion recovery MRI images; T1 = 2D axial non-enhanced T1-weighted spin-Echo or 3D non-enhanced T1-weighted gradient-Echo MRI images; Gd T1 = Gadolinium-enhanced T1-weighted spin-Echo MRI images; r_T1 = registered 2D axial non-enhanced T1-weighted spin-Echo or 3D non-enhanced T1-weighted gradient-Echo MRI images; r_Gd T1 = registered Gadolinium-enhanced T1-weighted spin-Echo MRI images; ROI = regions of interest; NAWM = normal appearing white matter; NAGM = normal appearing grey matter; ADC = apparent diffusion coefficient; CBF = cerebral blood flow; CBV = cerebral blood volume; MTT = mean transit time

The process management system named *Diffusion/Perfusion Project* (DPP) described in the paper is an in-house developed suite written in Matlab (The MathWorks, Natick, MA, USA) and requiring the Image Processing Toolbox. The DPP is a collection of software modules all related to MRI data management, sharing the same GUI (Graphical User Interface) and exchanging data with each other. DPP Suite integrates commonly used software tools which are able to perform different types of data analysis and management. The primary aim of DPP Suite is to create a uniform environment where it can be possible to assess a considerably larger number of data from MS patients compared to the other current analysis methods, reducing as much as possible analysis complexity, time required and potential human errors. The most relevant procedures manageable through DPP Suite are schematically presented in Fig. 1, where data and operations are shown as a flowchart. In summary, DPP Suite operations include: 1) image pre-processing; 2) registration of T1-weighted images; 3) lesion segmentation and classification; 4) registration of lesion masks; 5) normal appearing tissue segmentation; 6) PWI and DWI map generation; 7) registration of tissue segmentation and quantitative MRI maps; 8) data analysis.

### Pre-processing

Image pre-processing starts with the automatic anonymization of MRI sequences for each patient which are exported from a PACS database and copied in a local repository dynamically linked with the DPP Suite. This process aims at protecting patient identity during the whole procedure. Furthermore, demographic data, resulting analysis and patient identity are stored as a protected file controlled by the DPP Suite. The anonymization procedure is performed by Image Processing Toolbox Matlab function with some adaptations. The original embedded task for data anonymization, called *dicomanon*, was modified generating a function called *dpp_dicomanon* that can strengthen the patient data confidentiality level if needed. After anonymization, DICOM format [27] files are converted in NIFTI format [28] using the Statistical Parametric Mapping (SPM) 8 toolbox (http://www.fil.ion.ucl.ac.uk/spm/) [29]. Notably, pre-processing is performed on a limited dataset that includes the following MRI sequences: axial fluid-attenuated inversion recovery (FLAIR); two-dimensional (2D) axial non-enhanced T1-weighted spin-echo or three-dimensional (3D) non-enhanced T1 gradient-echo; axial Gadolinium (Gd)-enhanced T1-weighted spin-echo; axial DWI and axial PWI. Therefore, before data conversion, a DPP function selects patients having all MRI sequences requested for analysis, excluding those with incomplete radiological data. Concurrently, a set of report files containing a checklist of unavailable sequences for each non-conforming patient is generated. In addition, all MRI data are reorganized in a storage structure aimed at making a uniform data format, including sequences names, filenames or data storing and other details, that is independent of that given by different MRI scanners (e.g. Philips, Siemens or GE), without modifications in information content.

### Registration of T1-weighted images

**Fig. 2:**
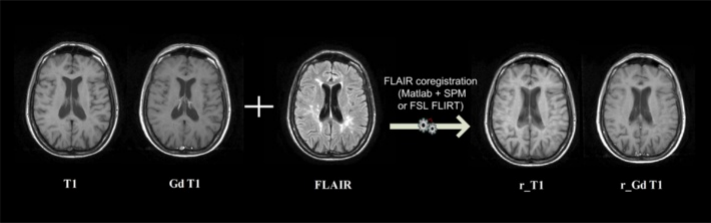
An illustrative example of registration of T1-weighted images. SPM = Statistical Parametric Mapping; FSL = FMRIB Software Library; FLIRT = FMRIB’s Linear Image Registration Tool; FLAIR = axial fluid-attenuated inversion recovery MRI images; T1 = 2D axial non-enhanced T1-weighted spin-Echo or 3D non-enhanced T1-weighted gradient-Echo MRI images; Gd T1 = Gadolinium-enhanced T1-weighted spin-Echo MRI images; r_T1 = registered 2D axial non-enhanced T1-weighted spin-Echo or 3D non-enhanced T1-weighted gradient-Echo MRI images; r_Gd T1 = registered Gadolinium-enhanced T1-weighted spin-Echo MRI images

In this first step (Fig. 2), both axial 2D non-enhanced T1-weighted spin-echo or 3D non-enhanced T1 gradient-echo and axial Gd-enhanced T1-weighted spin-echo are registered in FLAIR space using an automated process based on the employment of a DPP module invoking one of the two following external software packages: SPM or FMRIB’s Linear Image Registration Tool (FLIRT) from FMRIB Software Library (FSL) suite (http://fsl.fmrib.ox.ac.uk/fsl/fslwiki/flirt) [30, 31]. Although T2-weighted images are generally considered the most sensitive in detecting infratentorial lesions [32], FLAIR images serve for the following lesion segmentation since they improve lesion/brain contrast due to the suppression of cerebrospinal fluid signal [33]. On the other hand, T1-weighted images allow the identification of MS T1 hypointense (black holes) and T1 Gd-enhancing lesions as well as the evaluation of brain atrophy [17]. In this regard, it is well-accepted that 3D are superior to 2D T1 images in the determination of brain volume [34]. However, we chose to process also 2D T1 images since, in clinical practice, not all centers are equipped to routinely perform 3D T1 sequences in MS patients. The choice between SPM and FSL FLIRT is user selectable.

### Lesion segmentation and classification

The development of automated techniques for lesion detection is one of the most central challenges in MS research. Therefore, a number of approaches have recently been proposed [21–23]. However, it is generally accepted that there are no automatic lesion segmentation methods with 100 % reliability [18, 19]. This is the reason why a series of semi-automated algorithms were designed and tested in order to support and simplify lesion segmentation and classification. This step is one of the most critical and complex because it involves both human operation and automated processes. In fact, two basic operations are implemented: a) semi-automatic lesion segmentation; b) semi-automatic lesion classification.

### Semi-automatic lesion segmentation

**Fig. 3:**
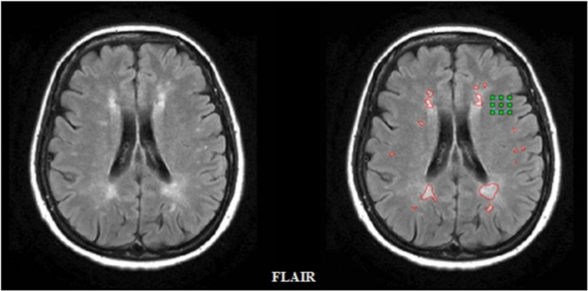
A descriptive example of semi-automated lesion segmentation process on fluid-attenuated inversion recovery (FLAIR) images. Segmented hyperintense lesions in red; area used for NAWM mean intensity estimation in green

As depicted in Fig. 3, this step is performed on FLAIR images using Jim (Jim 6.0, Xinapse Systems, Leicester, UK; http://www.xinapse.com) [34] as an external software package. After visual identification, each lesion is semi-automatically segmented by a local threshold-based technique. In addition, a region of interest (ROI) of the NAWM is manually outlined. The output of this process is an ROI file corresponding to the delineated areas.

### Semi-automatic lesion classification

**Fig. 4:**
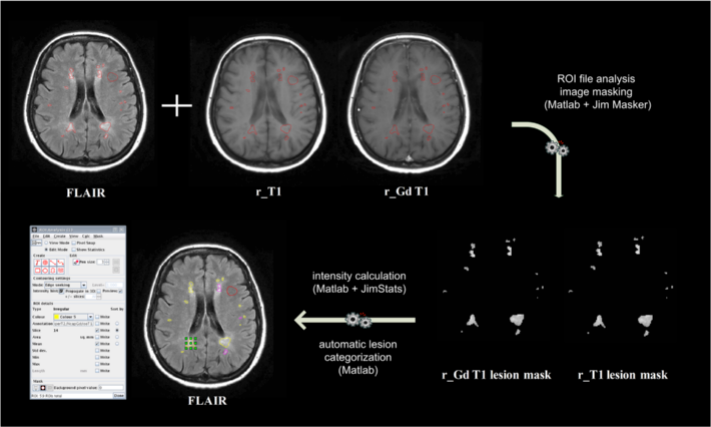
A demonstrative example showing the automatic lesion classification process. FLAIR = axial fluid-attenuated inversion recovery MRI images; r_T1 = registered 2D axial non-enhanced T1-weighted spin-Echo or 3D non-enhanced T1-weighted gradient-Echo MRI images; r_Gd T1 = registered Gadolinium-enhanced T1-weighted spin-Echo MRI images. ROI = Region Of Interest; area used for NAWM mean intensity estimation in red

Using the FLAIR-derived lesion ROI file, DDP Suite automatically masks the non-enhanced and Gd-enhanced T1-weighted images (Fig. 4). The DPP Suite then calculates the intensity of each lesion in both non-enhanced and Gd-enhanced T1-weighted sequence. Based on the recently proposed classification [20], the algorithm categorizes each T2-weighted hyperintense lesion, based on its intensity in four classes: 1) Gd-enhancing and T1-weighted isointense (C1); 2) Gd-enhancing and T1-weighted hypointense (C2); 3) non Gd-enhancing and T1-weighted isointense (C3); 4) non Gd-enhancing and T1-weighted hypointense, i.e. black holes (C4). As, in absence of serial MRI examinations, lesion activity is demonstrable only by Gd enhancement [35], C1 and C2 type lesions are considered as acute, whereas C3 and C4 lesions are judged as chronic [36, 37]. The output of the process is a recombined ROI file enriched with classification of each lesion given by a different color code for each lesion type and a descriptive text label where the type and characteristics of the single lesion are briefly reported (Fig. 4). Lesion classification is accomplished using a DPP algorithm based on a lesion intensity comparison to the NAWM ROI intensity level. The thresholds to identify the lesion classes are defined as a configurable multiplier of the standard deviation of the intensity values in the NAWM ROI.

1 $$ Thr{e}_{High}={\overline{I}}_{default}+Mf\cdot St-dev\left({I}_{NAWM}\right) $$

2 $$ Thr{e}_{Low}={\overline{I}}_{default}-Mf\cdot St-dev\left({I}_{NAWM}\right) $$

**Fig. 5:**
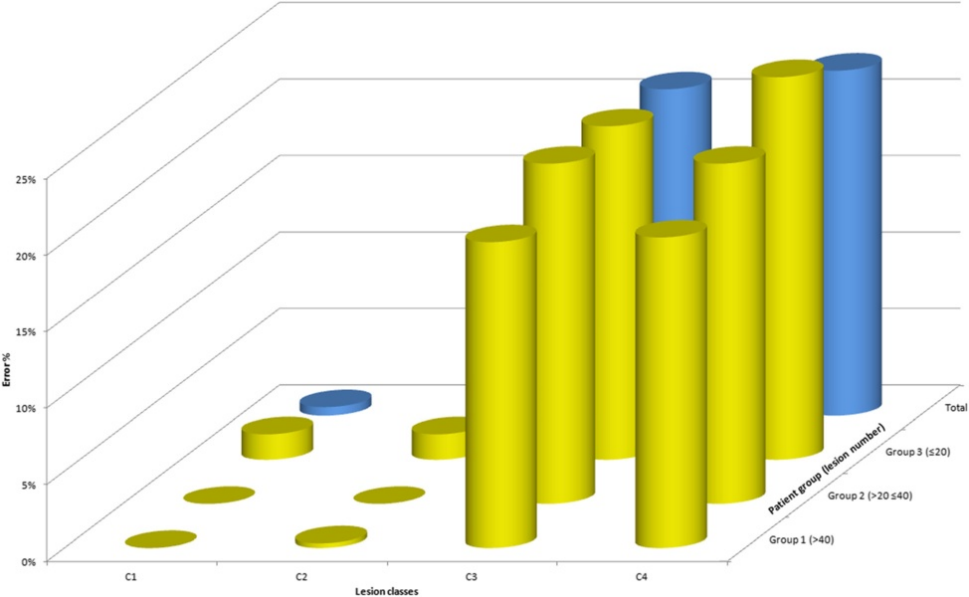
Automatic lesion classification algorithm performance for a group of 15 randomly selected patients. The automatic classification algorithm is compared with classification of the same lesions performed visually by an expert. Vertical axis shows the error percentage over total number of lesions. Group 1 (>40) = patient group 1 was composed by 5 patients with more than 40 lesions; Group 2 (>20 ≤ 40) = patient group 2 was composed by 5 patients with more than 20 and less than 40 lesions; Group 3 (≤20) = patient group 3 was composed by 5 patients with less than 20 lesions; Total = mean values between group 1, 2 and 3; C1 = lesion class 1; C2 = lesion class 2; C3 = lesion class 3; C4 = lesion class 4

Where *Ī* is the mean intensity of the NAWM and *Mf* is the multiplier factor and *St_dev* is the standard deviation calculation function. The mean intensity of ach lesion is then calculated and compared with the two thresholds referred to in formulas (1) and (2) that are calculated for both non-enhanced and Gd-enhanced T1-weighted images. The automatic lesion classification algorithm performance is briefly presented in Fig. 5 to document the accuracy of the procedure. The error percentage of automatic classification was less than 1 % for C1 and C2 lesion classes and about 21 % and 23 % for C3 and C4 lesion classes, respectively. It is important to underline that the low error percentage found for C1 and C2 lesion classes could be related to the small number of this type of lesions occurred in the selected patients, as well as in all patients analyzed. Therefore, these results support the need of a visual correction for automatic lesion classification. The output of the automatic lesion classification process is a text file in Jim ROI Analysis Tool format, containing all the ROI definitions and classifications. Automatic lesion classifications are visually checked and revised by the operator. The new checked file is then automatically reloaded and converted by DDP Suite into different revised ROI files, including the lesions categorized as total lesions, lesion classes (C1, C2, C3 and C4) and single lesion, which are then used in the following steps.

### Registration of lesion masks

**Fig. 6:**
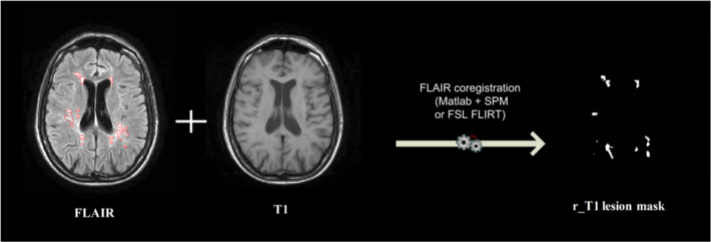
A graphic example of registration of lesion masks. FLAIR = axial fluid-attenuated inversion recovery MRI images; T1 = 2D axial non-enhanced T1-weighted spin-Echo or 3D non-enhanced T1-weighted gradient-Echo MRI images; SPM = Statistical Parametric Mapping; FSL = FMRIB Software Library; FLIRT = FMRIB’s Linear Image Registration Tool; r_T1 = registered 2D axial non-enhanced T1-weighted spin-Echo or 3D non-enhanced T1-weighted

This stage requires the creation of a lesion mask in the T1 space and is performed by SPM or FSL FLIRT external software packages invoked by a DPP module. Briefly, FLAIR images previously segmented are used to automatically produce a lesion mask that is coregistered in the non-enhanced T1-weighted space to obtain a registered lesion mask. When FLIRT is chosen, coregistration is done applying to the lesion mask in the FLAIR space the transformation matrix generated by the registration of FLAIR and T1. If SPM is selected, T1 is set as the reference image, FLAIR as source and the lesion mask as other. The result is a black and white lesion mask that is a binarization of the brain lesion area where lesions are white and the remaining brain tissue is black (Fig. 6).

### Normal appearing tissue segmentation

**Fig. 7:**
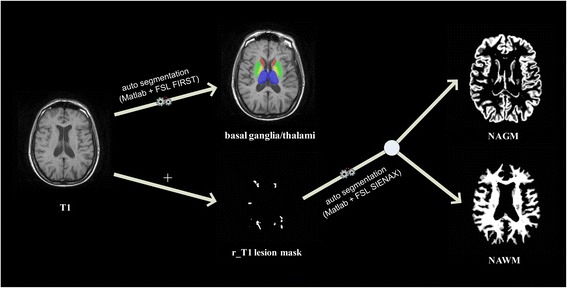
An illustrative example of normal appearing tissue segmentation. T1 = 2D axial non-enhanced T1-weighted spin-Echo or 3D non-enhanced T1-weighted gradient-Echo MRI images; FSL = FMRIB Software Library; FIRST = FMRIB’s Integrated Registration and Segmentation Tool; SIENAX = Structural Image Evaluation using Normalization of Atrophy; r_T1 = registered 2D axial non-enhanced T1-weighted spin-Echo or 3D non-enhanced T1-weighted gradient-Echo MRI images; NAGM = normal appearing grey matter; NAWM = normal appearing white matter

As depicted in Fig. 7, DPP Suite invokes automatic segmentation of the basal ganglia and thalami via the external FSL tool FMRIB’s Integrated Registration and Segmentation Tool (FIRST) (http://fsl.fmrib.ox.ac.uk/fsl/fslwiki/first) [38]. The input is the non-enhanced T1-weighted sequence. The segmented basal ganglia structures include the right and left caudate, putamen and globus pallidus. The following step is represented by the automatic segmentation of grey and white matter for which DPP Suite invokes FSL SIENAX (Structural Image Evaluation using Normalization of Atrophy) (http://fsl.fmrib.ox.ac.uk/fsl/fslwiki/SIENA) [39]. In this case, the inputs are non-enhanced T1-weighted sequence and registered T1 lesion mask, whereas the output are two files including NAGM and NAWM images obtained after subtracting registered T1 lesion mask from non-enhanced T1-weighted. In addition, FSL SIENAX generates a report file including total brain tissue volume, as a whole and normalized according to skull size, and normalized NAGM and NAWM volumes.

### PWI map generation

PWI studies are performed with a dynamic susceptibility contrast (DSC) MRI first-pass bolus-tracking technique using echo-planar gradient-echo T2* sequences. PWI analysis provides relative measurements of brain hemodynamic parameters such as Cerebral Blood Flow (CBF), Cerebral Blood Volume (CBV) and Mean Transit Time (MTT). CBF, CBV and MTT maps are generated by a singular value decomposition (SVD) deconvolution operation based on the measurement of an arterial input function (AIF) [40]. The calculation of PWI maps is automatically performed by Jim Perfusion Analysis tool invoked by DPP Suite. DSC images consist of a time-series of sequences, one volume for each time point, which monitor the concentration of an injected paramagnetic contrast agent transiting from the blood vessels to the brain tissue. Therefore, AIF determination implies the knowledge of the exact volume where the contrast agent is perceptible in the brain (Contrast Arrival Point). For that reason, the DPP Suite implements an algorithm that identifies the contrast arrival volume number using Jim Stats. This algorithm evaluates the mean intensity in volumes, using in subsequent steps the average intensity of the previous analyzed volumes, in order to reduce the noise affecting the images. It detects when the difference of mean intensity between two subsequent volumes is more than an adjustable threshold. The threshold is not an absolute value or an absolute percent, but is a fraction of the difference between maximum and minimum intensity value measured in all volumes of the sequence. Thus, the threshold is less dependent from the volume intensity absolute level.

### DWI map generation

**Fig. 8:**
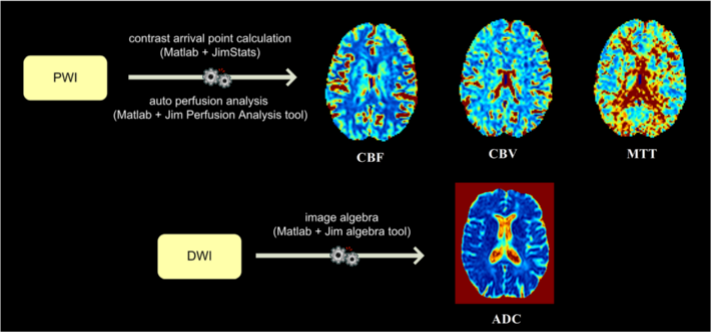
A descriptive example of generated apparent diffusion coefficient (ADC) and perfusion-weighted imaging (PWI) maps. CBF = Cerebral Blood Flow; CBV = Cerebral Blood Volume; MTT = Mean Transit Time; DWI = Diffusion Weighted Imaging

DWI studies are often performed with a single-shot echo-planar T2 spin-echo sequence according to the Stejskal-Tanner method [41]. The diffusion gradients are applied in three orthogonal directions (x, y, z) with two b-values (0 and 1000 s/mm^2^) to form the isotropic DWI images at b 1000 s/mm^2^. DWI analysis takes as input T2 images and extracts from these images apparent Diffusion Coefficient (ADC) maps, related to each Cartesian axis. More precisely, as reported elsewhere [42], ADC maps are generated using T2-weighted images at b 0s/mm^2^ and isotropic DWI images at b 1000 s/mm^2^ obtained in all three orthogonal directions. All calculations are automatically performed by Jim Image Algebra tool invoked by DPP Suite controlling the entire process. The process output is an average ADC map, obtained calculating the mean of the previously calculated ADC on the three Cartesian axes. After this process, previously generated ADC maps and PWI maps are ready to be registered in the next step (Fig. 8).

### Registration of tissue segmentation and quantitative MRI maps

This step is aimed at automatically translating the results of all intermediate processes previously described in the FLAIR space to allow the final data analysis in which DWI and PWI values are measured in every lesion as well as in the basal ganglia and thalami, NAGM and NAWM. DPP Suite invokes SPM or FSL FLIRT external software tools which generate the following registered maps:

From T1 space:

1. Basal ganglia and thalami maps;
2. NAGM map;
3. NAWM map;
  from DWI space:
4. Average ADC map;
  from PWI space:
5. CBV, CBF and MTT maps.

**Fig. 9:**
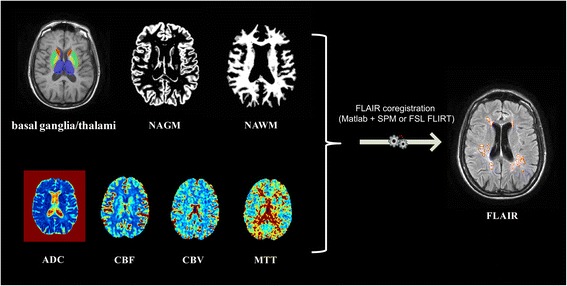
A graphic example of registration of tissue segmentation and quantitative MRI maps. NAGM = normal appearing grey matter; NAWM = normal appearing white matter; ADC = apparent diffusion coefficient; CBF = cerebral blood flow; CBV = cerebral blood volume; MTT = mean transit time; SPM = Statistical Parametric Mapping; FSL = FMRIB Software Library; FLIRT = FMRIB’s Linear Image Registration Tool; FLAIR = axial fluid-attenuated inversion recovery MRI images

The different maps are registered into the FLAIR space and used in the next data analysis (Fig. 9). The coregistration is performed using a similar method as in registration of lesion masks.

### Data analysis

**Fig. 10:**
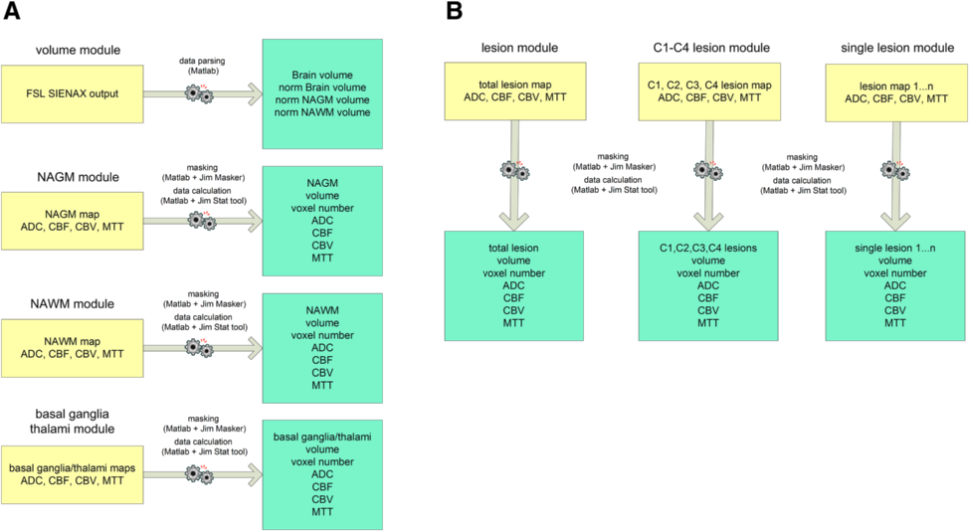
Two diagrams describing DPP data analysis. Panel **a**: measurements of total, brain normal appearing grey matter (NAGM) and normal appearing white matter (NAWM) volume and voxel number, both as a whole and normalized according to skull size, and measurements of apparent diffusion coefficient (ADC), cerebral blood flow (CBF), cerebral blood volume (CBV) and mean transit time (MTT) in basal ganglia, NAGM and NAWM. Panel **b**: measurements of apparent diffusion coefficient (ADC), cerebral blood flow (CBF), cerebral blood volume (CBV) and mean transit time (MTT) in previously saved and stored FLAIR lesion categorized as total, class C1, C2, C3, C4 and single. norm = normalized; C1 = enhancing T2-weighted hyperintense and T1-weighted isointense; C2 = enhancing T2-weighted hyperintense and T1-weighted ipointense ; C3 = non-enhancing T2-weighted hyperintense and T1-weighted isointense; C4 = non-enhancing T2-weighted hyperintense and T1-weighted ipointense, black holes

The final step represents the key advance performed by the DPP Suite compared to existing semi-automated methods. Measurements are obtained by the integration of the entire set of images and maps previously generated. This result is obtained using distinct software modules. In this stage, two groups of automatic analysis are developed. In the first one (Fig. 10, panel a), NAGM, NAWM and basal ganglia maps are used to mask ADC and PWI maps owing to Jim Masker invoked by DPP Suite. At this point, DPP Suite activates the Jim Stats tool that is able to calculate ADC, CBF, CBV and MTT values in these brain areas. In addition, the Jim Stats tool also measures NAGM, NAWM and basal ganglia/thalami volumes and voxel number and DPP Suite parses previously saved FSL SIENAX report file including whole and normalized total brain tissue volume and normalized NAGM and NAWM volumes. In the second group of data analysis (Fig. 10, panel b), DPP Suite invokes Jim Masker to mask DWI and PWI maps with FLAIR segmented lesions which DPP Suite has previously saved and stored as total, single class (C1, C2, C3 and C4) and single lesion maps. In this way, it is possible to obtain ADC, CBF, CBV and MTT values in all types of lesions where, in addition, also volume and voxel number are measured. All of these data are stored in a comma separated values (csv) file.

### Graphical User Interface

**Fig. 11:**
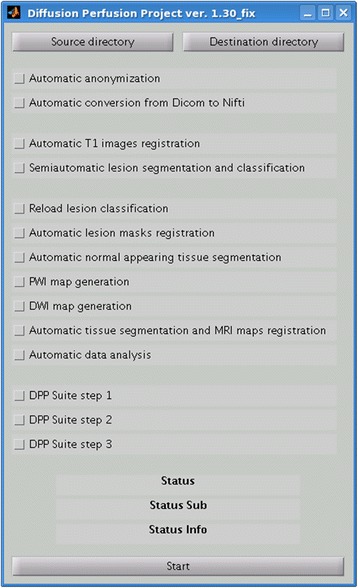
A picture illustrating the Graphical User Interface (GUI)

The Graphical user Interface (GUI) is shown in Fig. 11. The GUI keeps hidden all configuration parameters. Thus, the user is unable to modify the system parametrization and has a limited number of operations to perform. The configuration parameters are collected in a text configuration file. However, the configuration can be modified as needed by an expert operator, based on the needs of the study. These parameters are not shown in the GUI to facilitate the use of DPP suite by non-expert operators. The GUI configuration presents two sections: the first area in which all the process steps are singularly selectable and the second area where only three process macro steps can be activated. All these steps are performed automatically by DPP Suite, except lesion segmentation and classification which requires human intervention. The GUI presents also two directory browsing selection buttons for a more user friendly and flexible selection of input and output data directories. In order to control the process status and evolution, three status bars are also present in the lower part of the GUI window: a) a status bar defining the step under execution or the process execution status; b) a status-sub bar describing the sub-module under execution; c) a status-info bar indicating the patient number whose data are under analysis. The lower button is the step/process activation button, which starts the selected operations to be executed in an automated way by the DPP Suite.

### Process safety and data security

**Fig. 12:**
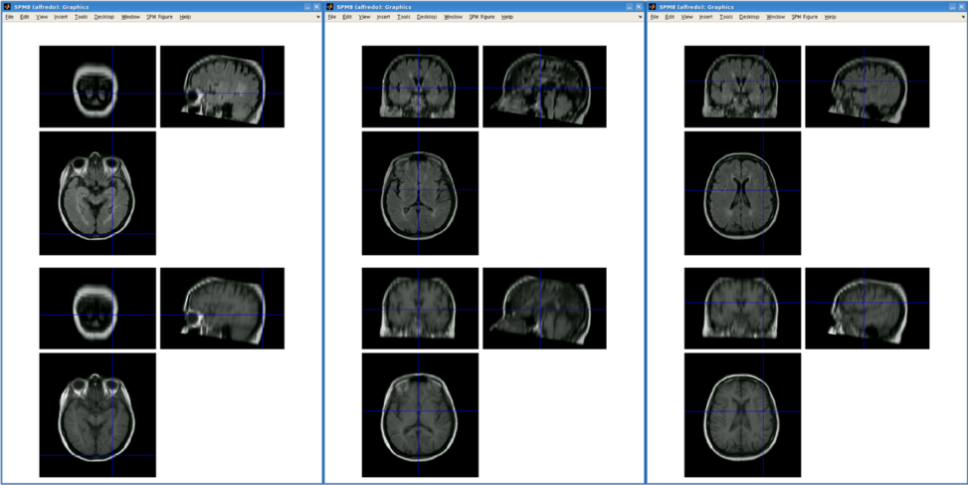
A visual coregistration check example using Check Reg tool in SPM. Left to right are presented three panels showing the output of Check Reg in case of a T1 (lower images) to FLAIR (higher images) sequences coregistration. Three relevant points were selected in order to check coregistration visually

All process steps, such as utilized parameters, the final results and the intermediate data are logged, saved and stored in the DPP Suite. This ensures complete environment preservation. For example, when an external tool is invoked by the DPP Suite both images and results and the tool’s standard output/error data log is read and saved. This approach allows post processing visual verification of e.g. co-registration (SPM or FSL FLIRT), tissue segmentation (SIENAX or FSL FIRST) or automatic detection of AIF-like voxels (Jim Perfusion tool). In Fig. 12 is presented an example of coregistration check performed using the Check Reg tool part of SPM module for functional MRI. Although processing parameters were tuned and optimized for every single step in DPP suite, in case of failure, configuration can be modified to correct processing errors. In this way, a clinician can check the results and make sure that they are accurate. Other security measures are integrated in DPP suite as well. For example, if a set of data represent both an output for the *(n-1)th* step and the input for the *(n)th* step, the entire data are copied before being modified by the *(n)th* step. Moreover, execution times and software version are preserved for every run of the suite (e.g. pre-processing data structure). This approach allows data reconstruction, process verification and software debugging, making it possible to reproduce every single step or the entire process and, thus, future validation of the DPP Suite.

## Results

### Software metrics

**Fig. 13:**
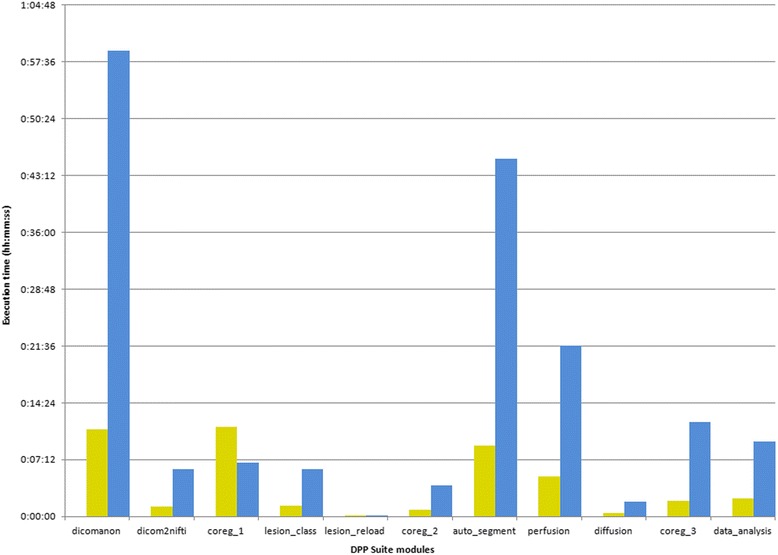
Execution time benchmark in a single patient (yellow/left) and 5 patients (blue/right). Vertical axis shows the execution times in the *hours:minutes:seconds* format. Matlab R2010a, Xinapse Jim 6.0, FMRIB Software library (FSL) 4.1.9 and Statistical Parametric Mapping (SPM) 8 were the software systems employed. coreg_1 = registration of T1-weighted images; lesion_class = lesion classification; coreg_2 = registration of lesion masks; auto_segment = normal appearing tissue segmentation; coreg_3 = registration of tissue segmentation and quantitative MRI maps

In order to evaluate the DPP Suite performance, the hardware and software configurations are listed in Table 1. It is important to note that the entire suite is carried out by a Linux Virtual Machine running in a Windows based PC. The execution time for all modules in a single patient (woman; 33 year old; clinically and MRI inactive with Relapsing-Remitting MS; disease duration = 84 months; Expanded Disability Status Scale = 2.5) and in five patients (4 women and 1 men; mean age = 46.4 ± 3.7 years; 2 clinically and MRI active and 3 clinically and MRI inactive with Relapsing-Remitting MS; median disease duration = 1 months; median Expanded Disability Status Scale = 1.5) were evaluated to test the DPP Suite performance. This analysis resulted linear regarding the number of patients, while the execution time varied as a function of number of lesions of patients (Fig. 13). Typical execution time for a single patient can vary from 30 to 55 min depending on the patient characteristics (number of lesions and MRI quality). The key information derived from this analysis is that the most time-consuming process is the anonymization with *dpp_dicomanon*. Interestingly, the typical execution time of the entire suite analysis is relatively short (45 min), which seems to be acceptable for a research use. It is important to note that the DPP Suite is able to work with different equipment since it can run on all hardware supporting VMware player (https://www.vmware.com/) or compatible virtualization software.

**Table 1:** Test Bench Hardware and Software

Hardware Host PC	
CPU	Intel Core i7-4770
Ram	8 Gb
Hard Disk	SSD + Raid 1 disk
Software Host PC	
Operating System	Windows 7 professional
Virtualization	VMware player 6.0.3
Software Guest PC	
Operating System	Scientific Linux 5.5

*CPU* Central Processing Unit, *SSD* Solid State Drive, *Raid* Redundant array of independent disks

### Reproducibility of results

The repeatability of data coming from DPP Suite was tested with the analysis of lesion number and ADC, CBF, CBV and MTT values obtained from 38 patients with Relapsing-Remitting MS (31 women and 7 men; mean age = 45.8 ± 9.1 years; 8 clinically active and 30 clinically inactive; median disease duration = 9 months; median Expanded Disability Status Scale = 2.0) by two different readers (EG with 2 years of experience; EF with 15 years of experience) who performed a double blinded, independent semi-automatic lesion segmentation and classification of T2-weighted hyperintense lesions. After checking data for normality by using the Kolmogorov-Smirnov test, continuous variables were compared using Mann-Whitney U test or independent-samples t test when appropriate. In addition, kappa inter-observer agreement was calculated. Kappa values were interpreted according to the proposed standards of Landis and Koch: 0–0.20 (slight agreement); 0.21–0.40 (fair agreement); 0.41–0.60 (moderate agreement); 0.61–0.80 (substantial agreement); 0.81–1.00 (almost perfect agreement) [43]. Tables 2 and 3 show that there were not statistically significant differences between the two readers in the evaluation of lesion number and ADC, CBF, CBV and MTT values detected in the four classes of T2-weighted hyperintense lesions. Inter-observer agreement between the two readers was substantial for lesion number and ADC, CBF, CBV and MTT values in C1 lesion class and for lesion number and ADC and MTT values in C2 lesion class, almost perfect for all parameters assessed in C3 and C4 lesion classes, and only moderate for CBF and CBV values in C2 lesion class. These findings argue for a good reproducibility of results provided by DPP Suite. On the other hand, the discrepancies observed between the two readers could be mainly attributable to differences in how the users have outlined and categorized the lesions, even if the selection of co-registration (FSL FLIRT versus SPM) and decision of whether an automated analysis passes quality control or not could represent other potential explanations. The modest inter-observer agreement between the two readers found for CBF and CBV values in C2 lesion class could be also affected by the current limitations in discriminating between acute active and chronic active Gd-enhancing and T1-weighted hypointense T2-weighted hyperintense lesions [44]. In any case, such differences can be minimized based on having some type of training between the users (i.e. which option should be selected and how to assess the quality of the analysis).

**Table 2:** Number of focal lesions and ADC, CBF, CBV and MTT values in C1 and C2

	C1 lesions				C2 lesions			
	First reader	Second reader	*p* value*	Kappa value	First reader	Second reader	*p* value*	Kappa value
Lesion number (sum, median, IQR, mean ± SD, range)	4, 0, 0-0, 0.1 ± 0.3, 0-1	7, 0, 0-0, 0.2 ± 0.5, 0-2	*p* = 0.242	0.692	9, 0, 0-0, 0.3 ± 0.6, 0-2	12, 0, 0-0, 0.2 ± 0.7, 0-3	*p* = 0.148	0.614
ADC x 10-3 s/mm2 (median, IQR, mean ± SD, range)	0, 0-0, 0.1 ± 0.3, 0-0.9	0, 0-0, 0.1 ± 0.3, 0-0.9	*p* = 0.256	0.792	0, 0-0, 0.2 ± 0.4, 0-1.1	0, 0-0, 0.1 ± 0.3, 0-1.2	*p* = 0.146	0.721
CBF ml/100g/min (median, IQR, mean ± SD, range)	0, 0-0, 15.8 ± 50.2, 0-244.9	0, 0-0, 24 ± 59.3, 0-244.9	*p* = 0.2400	0.686	0, 0-0, 35.3 ± 76.2, 0-342.2	0, 0-0, 14.5 ± 39, 0-136.6	*p* = 0.108	0.450
CBV 100g/min (median, IQR, mean ± SD, range)	0, 0-0, 0 ± 0, 0-0.2	0, 0-0, 0 ± 0.1, 0-0.2	*p* = 0.248	0.742	0, 0-0, 0 ± 0.1, 0-0.4	0, 0-0, 0 ± 0.1, 0-0.3	*p* = 0.114	0.464
MTT seconds (median, IQR, mean ± SD, range)	0, 0-0, 0.8 ± 2.8, 0-15.3	0, 0-0, 1.1 ± 2.7, 0-11.2	*p* = 0.251	0.719	0, 0-0, 2 ± 3.8, 0-13.5	0, 0-0, 1.2 ± 3.3, 0-13.5	*p* = 0.136	0.673

Values obtained by two different readers in Gd-enhancing and T1-weighted isointense (C1) and Gd-enhancing and T1-weighted hypointense (C2) T2-weighted hyperintense lesions from 38 patients with Relapsing-Remitting Multiple Sclerosis. *ADC* Apparent Diffusion Coefficient, *CBF* Cerebral Blood Flow, *CBV* Cerebral Blood Volume, *MTT* Mean Transit Time, *First reader* EG, *Second reader* EF, *SD* Standard deviation, *IQR* Interquartile range, *Mann-Whitney

**Table 3:** Number of focal lesions and ADC, CBF, CBV and MTT values in C3 and C4

	C3 lesions				C4 lesions			
	First reader	Second reader	*p* value	Kappa value	First reader	Second reader	*p* value	Kappa value
Lesion number (sum, median, IQR, mean ± SD, range)	1075, 18, 5.5-33.8, 28.3 ± 30.8, 0-129	1138, 19, 5.5-38.8, 29.9 ± 32.6, 0-132	*p* = 0.446*	0.921	689, 11, 3-21.5, 18.1 ± 22.8, 0-99	623, 8, 2-20, 16.4 ± 20.3, 0-87	*p* = 0.420*	0.904
ADC x 10-3 s/mm2 (median, IQR, mean ± SD, range)	0.9, 0.9-1, 0.9 ± 0.2, 0-1.2	0.9, 0.9-1, 0.9 ± 0.2, 0-1.2	*p* = 0.350*	0.872	1, 0.9-1.1, 1 ± 0.2, 0-1.2	1, 0.9-1.1, 1 ± 0.2, 0-1.3	*p* = 0.448*	0.812
CBF ml/100g/min (median, IQR, mean ± SD, range)	131.6, 88.6-183.8, 137.5 ± 60.2, 0-295.9	139.3, 99.9-191.1, 141.8 ± 58.8, 0-295.9	*p* = 0.376**	0.844	124.3, 84.8-165.9, 124.6 ± 58.5, 0-263	105.5, 76.5-150, 115.1 ± 59.6, 0-270.8	*p* = 0.243**	0.848
CBV 100g/min (median, IQR, mean ± SD, range)	0.1, 0.1-0.2, 0.1 ± 0, 0-0.3	0.1, 0.1-0.2, 0.1 ± 0, 0-0.3	*p* = 0.500**	0.824	0.1, 0.1-0.2, 0.1 ± 0.1, 0-0.3	0.1, 0.1-0.1, 0.1 ± 0.1, 0-0.3	*p* = 0.181**	0.769
MTT seconds (median, IQR, mean ± SD, range)	8.3, 6.9-9, 8 ± 2.2, 0-12.1	8.1, 6.6-8.6, 7.8 ± 2.2, 0-12.1	*p* = 0.384**	0.836	8.4, 6.8-9.7, 8.2 ± 2.6, 0-15	8.4, 6.8-9.7, 81. ± 2.9, 0-15	*p* = 0.463**	0.828

Values obtained by two different readers in non Gd-enhancing and T1-weighted isointense (C3) and non Gd-enhancing and T1-weighted hypointense (C4) T2-weighted hyperintense lesions from 38 patients with Relapsing-Remitting Multiple Sclerosis. *ADC* Apparent Diffusion Coefficient, *CBF* Cerebral Blood Flow, *CBV* Cerebral Blood Volume, *MTT* Mean Transit Time, *First reader* EG, *Second reader* EF; *SD* Standard deviation, *IQR* Interquartile range, *Mann-Whitney; **t-test

Written informed consent was given by all patients before inclusion and the study design was approved by the Research Ethics Board of Azienda Ospedaliero-Universitaria di Ferrara (Italy) that is our Local Committee for Medical Ethics in Research

## Discussion and conclusions

In this work we implemented an alternative system to analyze DWI and PWI abnormalities in various classes of lesions and in normal appearing brain tissues from patients with MS. The novelty of our approach is represented by a semi-automated integration of several previously validated software packages which are sequentially activated instead of separately utilized. This has the effect of significantly reducing human intervention that can be a source of bias and improper tissue and/or lesion classification. This approach makes the analysis quicker, simpler and provides reliable results. In fact, except for two checkpoints represented by lesion segmentation and classification, DPP Suite procedures offer a highly automatic elaboration of a large amount of MRI data in which the different algorithms are harmonically and hierarchically incorporated. The main objective of the development of our DPP Suite is to provide clinical researchers with a practical and reproducible tool to clarify the actual significance of DWI and PWI disturbances in MS. On the other hand, the coherent integration process obtained with the DPP Suite offers an intuitive time improvement compared to manually performing the individual steps. Finally, the DPP Suite provides a “best of breed” approach for the external tool choice and usage for each specific operation, a software modularity leading to a better management of method complexity and reusability in the setting of process safety. However, the evaluation of the DPP Suite in terms of usability, sustainability and maintainability by using different target users remains to be addressed.
